# Characterization and Differentiation of the Tumor Microenvironment (TME) of Orthotopic and Subcutaneously Grown Head and Neck Squamous Cell Carcinoma (HNSCC) in Immunocompetent Mice

**DOI:** 10.3390/ijms22010247

**Published:** 2020-12-29

**Authors:** Matthias Brand, Simon Laban, Marie-Nicole Theodoraki, Johannes Doescher, Thomas K. Hoffmann, Patrick J. Schuler, Cornelia Brunner

**Affiliations:** Department of Otorhinolaryngology, University Hospital Ulm, Frauensteige 12, 89075 Ulm, Germany; simon.laban@uniklinik-ulm.de (S.L.); marie-nicole.theodoraki@uniklinik-ulm.de (M.-N.T.); johannes.doescher@uniklinik-ulm.de (J.D.); t.hoffmann@uniklinik-ulm.de (T.K.H.); patrick.schuler@uniklinik-ulm.de (P.J.S.); cornelia.brunner@uniklinik-ulm.de (C.B.)

**Keywords:** tumor microenvironment, head and neck cancer, SCC VII orthotopic mouse model, SCC VII subcutaneous mouse model, regulatory B cells, CD4^+^ T cells, adenosine-related immunosuppression, tumor-infiltrating lymphocytes

## Abstract

For the development and evaluation of new head and neck squamous cell carcinoma (HNSCC) therapeutics, suitable, well-characterized animal models are needed. Thus, by analyzing orthotopic versus subcutaneous models of HNSCC in immunocompetent mice, we evaluated the existence of adenosine-related immunosuppressive B- and T lymphocyte populations within the tumor microenvironment (TME). Applying the SCC VII model for the induction of HNSCC in immunocompetent C3H/HeN mice, the cellular TME was characterized after tumor initiation over time by flow cytometry. The TME in orthotopic grown tumors revealed a larger population of tumor-infiltrating lymphocytes (TIL) with more B cells and CD4^+^ T cells than the subcutaneously grown tumors. Immune cell populations in the blood and bone marrow showed a rather distinct reaction toward tumor induction and tumor location compared to the spleen, lymph nodes, or thymus. In addition, large numbers of immunosuppressive B- and T cells were identified within the TME but also in secondary lymphoid organs, independently of the tumor initiation site. The altered immunogenic TME may influence the response to any treatment attempt. Moreover, when analyzing the TME and other lymphoid organs of tumor-bearing mice, we observed conditions reflecting largely those of patients suffering from HNSCC suggesting the C3H/HeN mouse model as a suitable tool for studies aiming to target immunosuppression to improve anti-cancer therapies.

## 1. Introduction

### 1.1. Squamous Cell Carcinoma of the Head and Neck

Head and neck cancer (HNC) represents a heterogeneous group of tumors at different anatomical locations and is the sixth most common tumor disease worldwide, with an increase of over 650,000 new diagnoses per year [1]. More than 95% of HNC are squamous cell carcinomas (HNSCC) with a mortality rate of about 50% [2]. The main risk factors for the development of HNSCC are alcohol consumption, smoking, and infection with the human papillomavirus (HPV) [3]. In addition to the histological classification of head and neck carcinomas, their description and classification are also based on localization and the TNM stage (T = size of the original (primary) tumor, N = (regional) lymph nodes, M = distant metastasis). A definition of the HNSCC by histological tissue type, tumor size, and tumor spread determines the therapeutic concept for the patient. HNSCC treatment is mainly based on surgical-, radiotherapy-, and/or chemotherapy treatment options. Despite intensive research, new developments in modern medicine, and innovative therapeutic concepts, the 5-year survival rate has hardly improved in the last 20 years [4]. Besides these three therapy pathways, immunotherapy represents a fourth pillar in the therapy of HNSCC. The interaction of the immune system with HNSCC is currently a subject of intensive research. For the development and evaluation of new antitumor therapeutics, suitable animal models are needed that replicate the course of the disease and reflect the interaction of the tumor with the immune system of the patient.

### 1.2. Tumor Microenvironment and Immunosuppressive Mechanisms

The immunogenic microenvironment of tumors consists of numerous components, which together determine the fate of tumor cells. Besides tumor cells, a tumor also contains connective tissue, blood vessels, stromal cells, and immune cells. In this environment, anti-inflammatory mechanisms are contrasted with inflammatory, anti-tumoral processes. To evade detection by the immune system, tumor cells use various mechanisms to prevent, attenuate, or completely neutralize an immune response. In recent years, regulatory B cells (Breg) have been identified as a new immunosuppressive cell population [5]. By expressing the two ectonucleotidases CD39 and CD73 on their surface, Breg catalyze the degradation of ATP to immunosuppressive adenosine, and thus promote tumorigenesis. Among others, this mechanism is the subject of our research, and therefore a main focus while characterizing the immunogenic milieu of HNSCC in this work.

### 1.3. Immunotherapy Supports an Antitumoral Microenvironment

Restoring the immunological competence of patients is the aim of immunotherapy and a subject of intensive research. The discovery of immune checkpoint inhibitors such as Nivolumab leads to new therapeutic options, especially in HNSCC treatment [6]. Immune checkpoint inhibitors abrogate the suppressive function of inhibitor molecules, and hence, enable an enhanced response of the immune system. Over the last years, investigators have identified different immune cell populations within the tumor microenvironment to be affected by immunotherapy [5,7,8]. Previous work of our group revealed a critical role of B cells in tumor formation and therefore underlining B cells as a potential target for immunotherapy [9]. For further investigation, a reliable murine HNSCC model is needed that forms a physiologic tumor microenvironment. In this study, we compared two murine HNSCC models with different tumor induction sites to reveal any alterations which could cause adverse results in planed treatment studies.

### 1.4. Tumor Location Is Reported to Affect the Tumor Microenvironment

Preliminary studies by others have shown differences in the growth behavior of orthotopic tumors compared to ectopic tumor localizations in xenograft models [10]. Furthermore, the composition of the immunogenic milieu in tumor tissue was reported to be affected by the tumor localization. However, these studies mostly referred to certain immune cell types, and thus reflected a small part of the immune system. Studies characterizing the totality of all components of the tumor-induced immune response in mice are hardly available.

### 1.5. The C3H/HeN-SCC VII Model Represents an Immunocompetent HNSCC System

One of the available immunocompetent animal models for head and neck cancer research represents the injection of SCC VII cells into the mouse strain C3H/HeN [11]. Originally, a modification of this mouse strain was established for research on orthotopic HNSCC [12]. However, the C3H/HeJ mouse strain used in previous work has a homozygous TLR-4 mutation and is, therefore, considered immunodeficient. Preliminary studies showed altered tumor growth in C3H/HeJ mice compared to immunocompetent C3H/HeN mice [11,13]. The SCC VII cell line is a spontaneous tumor cell line of the head and neck region, cultivated from mice of the C3H strain [12]. The syngeneic C3H-SCC VII system is a widely used and accepted model for research on head and neck carcinomas [11,14,15,16]. The aggressively growing SCC VII cells are poorly immunogenic with a strong resistance to tumor-specific T cells [17]. In this study, we analyzed the tumor microenvironment (TME) of head and neck tumors induced in C3H/HeN mice. In addition, we evaluate this system for its applicability in proving newly established immunotherapeutic interventions through a comparison of the described situations in human HNSCC patients.

## 2. Results

### 2.1. Increased B Cell and CD4^+^ T Cell Numbers in the Orthotopic TME

**Figure 1:**
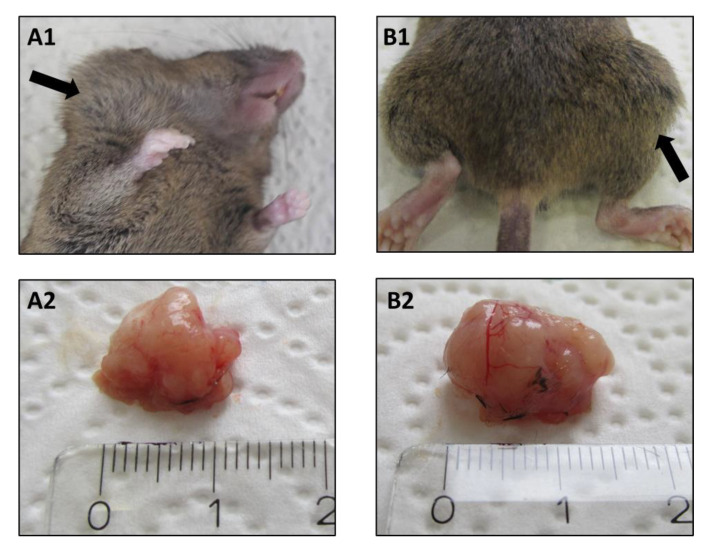
A representative example of an orthotopic tumor (**A1**,**A2**) and a subcutaneous tumor (**B1**,**B2**) grown for 21 days in a murine model (marked by black arrows). After preparation, both tumors are approximately 1 cm^3^ in size 21 days after tumor induction (**A2**,**B2**). The attached length unit indicates the size in centimeters (**A2**,**B2**). Macroscopically, good vascularization of the tumor can be seen (**A2**,**B2**).

**Figure 2:**
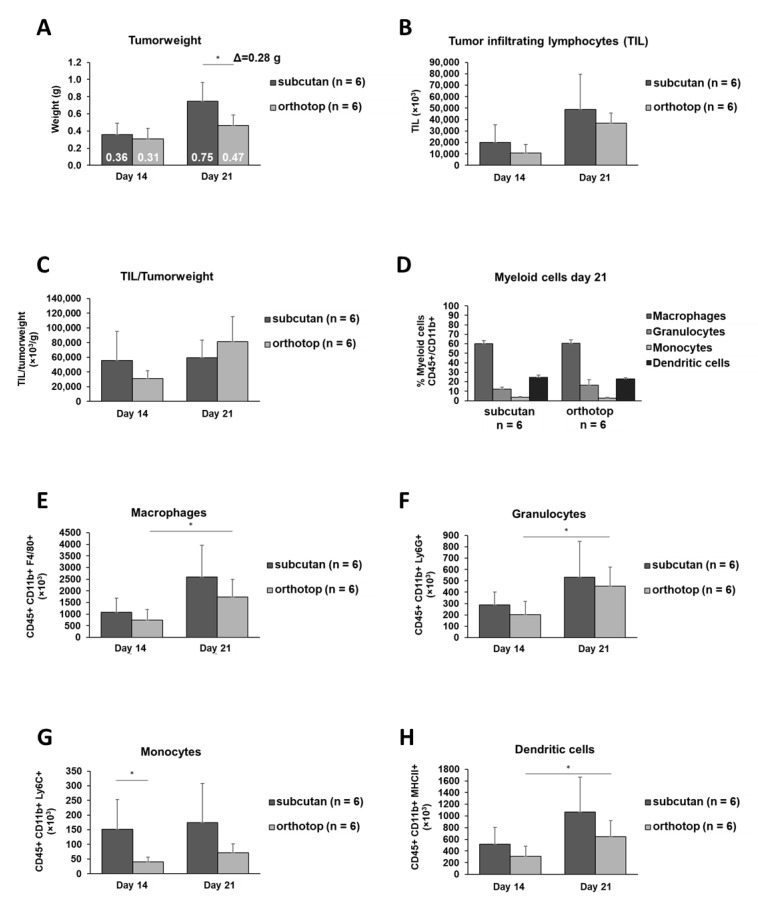
The tumor growth, the absolute number of tumor-infiltrating lymphocytes (TIL), the TIL/tumor weight, and the total number of myeloid cells of orthotopic and subcutaneous head and neck squamous cell carcinoma (HNSCC) bearing mice. Tumors were harvested 14 days and 21 days after tumor induction, and the tumor weight, including the difference in the average tumor weight of both experimental groups (Δ = 0.28g) (**A**), the absolute numbers of TIL (**B**), the numbers of TIL per g tumor (**C**), the relative TIL myeloid cell distribution (**D**), and the absolute numbers of macrophages, granulocytes, monocytes, and dendritic cells (**E**–**H**) were determined. On day 14 and day 21, the test group size for the subcutaneous group was *n* = 6, and for the orthotopic group *n* = 6. *p*-values < 0.05 were considered to be significant with (*). Data shown here are listed in Table S1.

In order to identify whether the site of tumor induction influences tumor growth and the TME, mice were randomly grouped, and SCC VII cells were injected either into the mouth floor or into the right flank (Figure 1). Twenty-one days after tumor induction, the subcutaneously grown tumor was significantly larger than the orthotopic grown tumor, with a weight difference of 0.28 g (Figure 2A), corresponding to an approximately 60% higher tumor mass compared to the orthotopic tumor. Despite the larger volume, the number of tumor-infiltrating lymphocytes (TIL) did not significantly differ between the two tumor models (Figure 2B). Remarkably, when the number of TIL was normalized to the tumor weight, a reversal of the prevalence of TIL in tumor tissue was observed. The number of TIL per tumor weight tended to be higher in the orthotopic grown tumor than in the subcutaneously grown tumor (Figure 2C). By further analysis of the TIL composition, this observation becomes more relevant. The relative distribution of myeloid cells is identical between both models 21 days after tumor induction (Figure 2D). Macrophages and dendritic cells represent the majority of myeloid cells in the TME (around 83%). Over time, while the number of monocytes remained equal in the TME, an increase in the absolute numbers of macrophages, granulocytes, and dendritic cells in both tumor models could be observed (Figure 2E–H). This increase is significant in the orthotopic grown tumor (macrophages *p* ≤ 0.033, granulocytes *p* ≤ 0.021, dendritic cells *p* ≤ 0.042).

**Figure 3:**
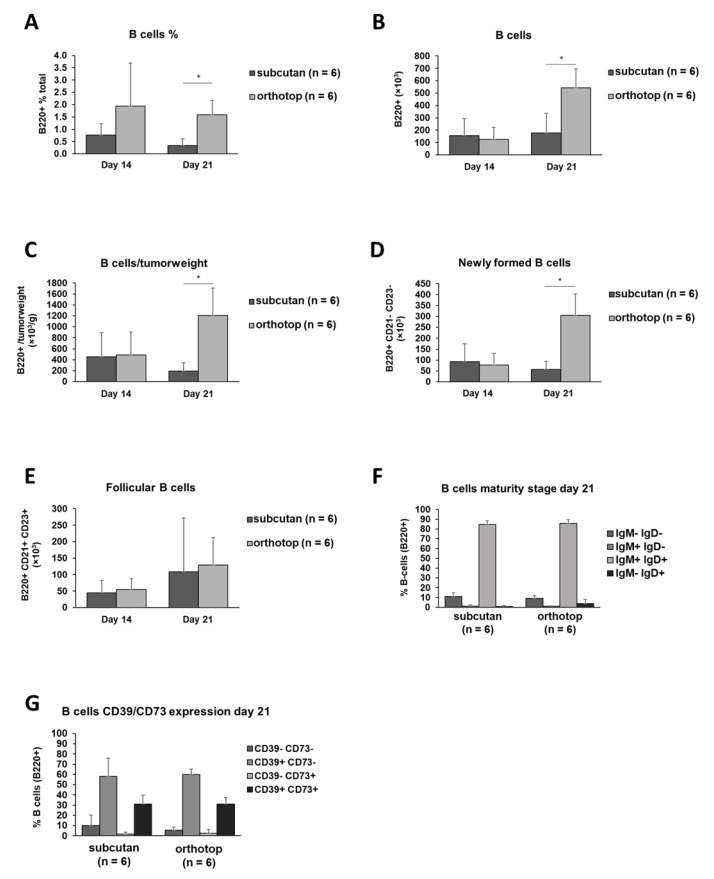
B cell populations in the tumor of orthotopic and subcutaneous HNSCC-bearing mice. (**A**–**C**) Tumors were harvested 14 days and 21 days after tumor induction, and TIL were isolated. TIL were analyzed by flow cytometry for B220^+^ B cell frequency. For further characterization, B cells were divided into newly formed B cells (**D**) and follicular B cells (**E**). Furthermore, the B cell population was characterized by their expression of IgM and IgD (**F**) as well as CD39 and CD73 (**G**). On day 14 and day 21, the test group size for the subcutaneous group was *n* = 6, and for orthotopic group *n* = 6. *p*-values < 0.05 were considered to be significant with (*). Data shown here are listed in Table S1.

Furthermore, we detected a significant increase in the total number (*p* ≤ 0.01) as well as in the frequency (*p* ≤ 0.005) of B cells in the orthotopic grown tumor (Figure 3A–C). By further analyzing B cell subpopulations, we revealed a significant increase in the number of newly formed B cells (*p* ≤ 0.002) within the orthotopic grown tumor while the number of follicular B cells stayed equal in both models (Figure 3D,E). The IgM and IgD as well as the CD39 and CD73 expression on the surface of those B cells, did not reveal any significant differences (Figure 3F,G). The majority of B cells in the TME express IgM and IgD (around 85% of B cells). Interestingly, we were able to observe a remarkable amount of B cells expressing CD39 and CD73 within both tumor models. Around 60% of TIL B cells express CD39 on their surface, and 30% of TIL B cells are CD39^+^/CD73^+^ double-positive (Figure 3G). Moreover, by checking the total number of B cells expressing both CD39 and CD73, we observed an increase of the CD39/CD73 positive B cell population during tumor formation over time at the expense of CD39^−^/CD73^−^ B cells (Figure S1), suggesting a role of the CD39^+^/CD73^+^ B cell population and the related adenosine mediated immunosuppressive pathway during tumorigenesis.

**Figure 4:**
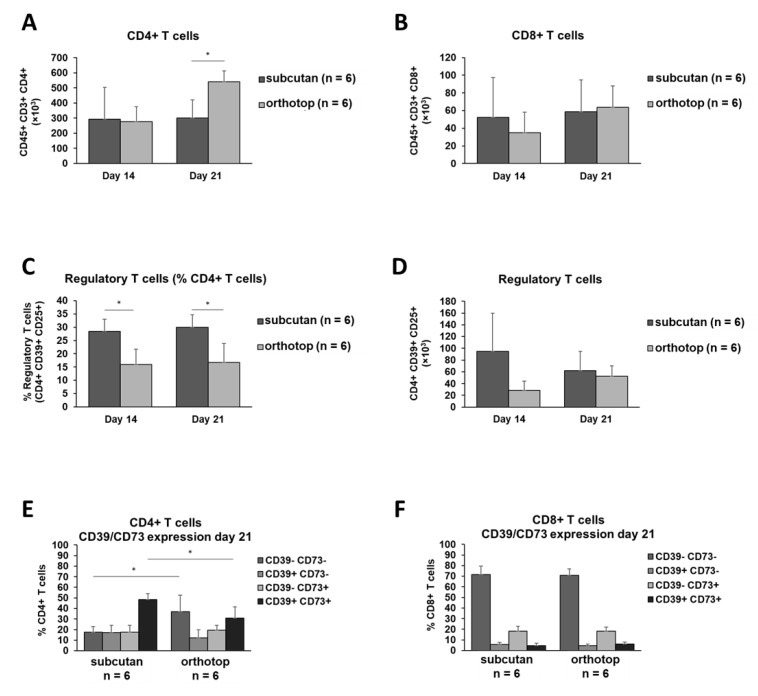
T cell populations in the tumor of orthotopic and subcutaneous HNSCC-bearing mice. (**A**–**D**) Tumors were harvested 14 days and 21 days after tumor induction, and populations of CD4^+^ and CD8^+^ T cells and regulatory T cells were analyzed using flow cytometry. (**E**,**F**) The expression profile for CD39/CD73 was determined on CD4^+^ and CD8^+^ T cells. On day 14 and day 21, the test group size for the subcutaneous group was *n* = 6, and for the orthotopic group *n* = 6. *p*-values < 0.05 were considered to be significant with (*). Data shown here are listed in Table S1.

Furthermore, we observed an increased population of CD4^+^ T cells within the orthotopic grown tumor 21 days after induction compared to the subcutaneously grown tumor (*p* ≤ 0.01, Figure 4A). The number of CD8^+^ T cells remained equal in both models during tumor formation (Figure 4B). Interestingly, compared to the subcutaneously grown tumor, a smaller percentage of CD4^+^ T cells in the orthotopic grown tumor expressed markers (CD4^+^ CD39^+^ CD25^+^) for a regulatory T cell phenotype (28% vs. 15%, *p* ≤ 0.01, Figure 4C). At the same time, the absolute number of those regulatory T cells in both tumor models did not show any differences (Figure 4D). The CD39 and CD73 expression on CD4^+^ T cells revealed a higher CD4-mediated immunosuppressive capability within the subcutaneously grown tumor 21 days after tumor induction (Figure 4E). The expression of both ectonucleotidases on CD8^+^ T cells did not show any differences (Figure 4F).

### 2.2. Tumor Formation Leads to an Increase in the Number of Leukocytes in the Blood of Tumor-Bearing Mice

**Figure 5:**
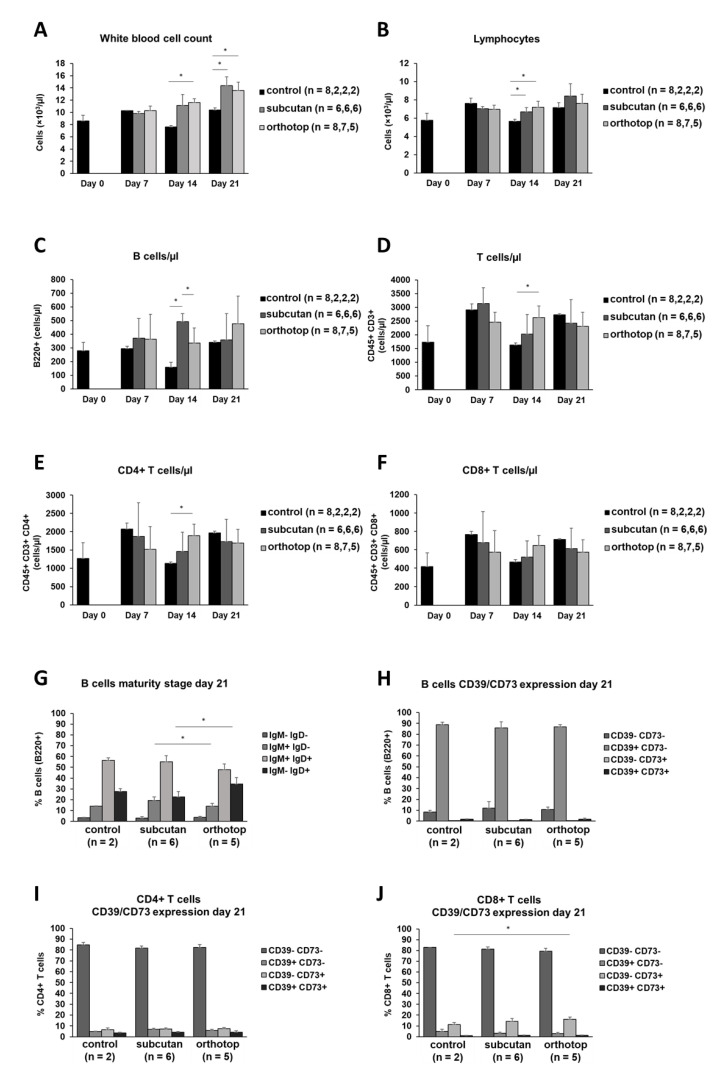
Cell populations in the peripheral blood of orthotopic and subcutaneous HNSCC-bearing mice. Leukocytes (**A**) and lymphocytes (**B**) were determined using an animal blood counter. (**C**–**F**) Populations of B cells and T cells were analyzed using flow cytometry. (**G**,**H**) The expression profile for IgM/IgD and CD39/CD73 was determined on peripheral B cells. (**I**,**J**) The expression profile for CD39/CD73 was determined on T cells. Cell numbers per µl blood were calculated based on the white blood cell count (WBC). Values are shown for day 0 (control), day 7, day 14, and day 21. On day 7, day 14, and day 21, the test group size for the subcutaneous group is *n* = 6, while for the control group *n* = 2. On day 0, the test group size for the control group is *n* = 8. The test group size for the orthotopic group on day 7 is *n* = 8, day 14 *n* = 7, and day 21 *n* = 5. *p*-values < 0.05 were considered to be significant with (*). Data shown here are listed in Table S2.

We observed an increase in white blood cells (leukocytes) in tumor-bearing mice. This increase was constant over time and most visible 21 days after tumor induction (Figure 5A). In contrast, the number of lymphocytes remained constant. There were smaller variations 14 days after tumor induction, but these were balanced out again during the course of the experiment (Figure 5B). Regarding the number of leukocytes or lymphocytes, there were no apparent differences between the two tumor models. Besides smaller alterations, the total number of B cells and T cells in the blood did not show any differences during tumor formation and was not altered by the tumor induction site (Figure 5C–F). The IgM and IgD expression as well as the CD39 and CD73 expression on B cells remained untouched by the tumor location and tumor formation. Nearly 90% of B cells in the blood expressed IgM, while being negative for IgD. Additionally, the majority of B cells were CD39- and CD73-negative (Figure 5G,H). Most of the T cells in the blood (>80%) did not show a CD39 or a CD73 expression (Figure 5I,J).

### 2.3. Splenic B and T Cells of Both Tumor Models Did Not Show Any Significant Differences

**Figure 6:**
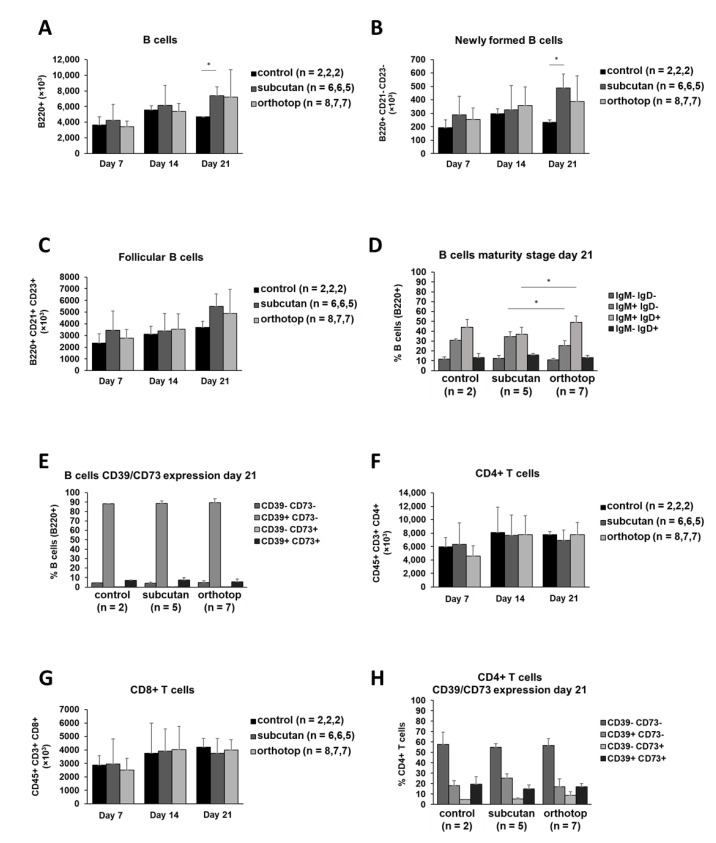

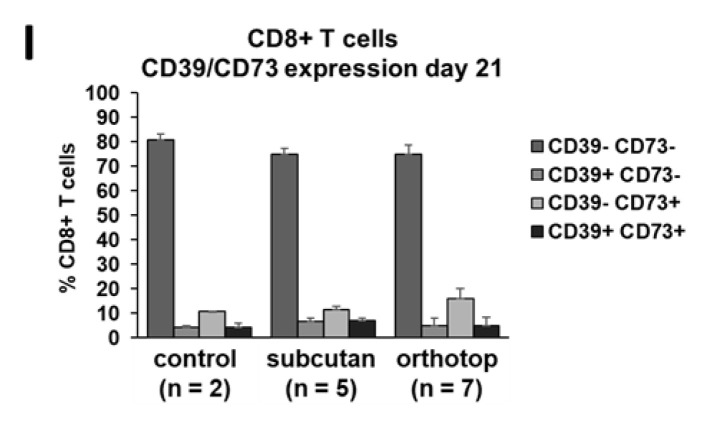
Cell populations in the spleen of orthotopic and subcutaneous HNSCC-bearing mice. (**A**–**C**) Populations of B cells and subgroups of B cells were analyzed using flow cytometry. (**D**,**E**) The expression profile for IgM/IgD and CD39/CD73 was determined on B cells. (**F**,**G**) Populations of CD4^+^ and CD8^+^ T cells were analyzed using flow cytometry. (**H**,**I**) The expression profile for CD39/CD73 was determined on T cells. Absolute cell numbers and percentages were calculated based on absolute and relative lymphocyte numbers. Values are shown for day 0 (control), day 7, day 14, and day 21. The test group size for the subcutaneous group is on day 7 is *n* = 6, day 14 *n* = 6, and day 21 *n* = 5. The test group size for the orthotopic group on day 7 is *n* = 8, day 14 *n* = 7, and day 21 *n* = 7. The test group size of the control group *n* = 2 on all days. *p*-values < 0.05 were considered to be significant with (*). Data shown here are listed in Table S3.

While the percentage of B cells within the total immune cell population in the spleen was equal in both tumor models during tumor formation (data not shown), the absolute number of B cells increased in tumor-bearing mice during tumor formation (Figure 6A). This increase was significant in subcutaneously grown tumors 21 days after tumor induction (*p* ≤ 0.04). This observation was also seen in B cell subpopulations, such as follicular B cells and newly formed B cells (Figure 6B,C). Regarding the IgM and IgD expression of splenic B cells, we were able to demonstrate an increase in IgM^+^ IgD^+^ B cells in the orthotopic model (around 50%) while the number of IgM^+^ IgD^−^ B cells was significantly reduced (*p* ≤ 0.015, Figure 6D). However, all of the described differences in the IgM and IgD expression on B cells occurred between the two tumor groups while there were no significant differences between the tumor groups and control group. The majority of approximately 85% splenic B cells express CD39 but not CD73 (Figure 6E). The T cell population in the spleen of tumor-bearing mice was not affected by tumor location (Figure 6F,G). In addition, the CD39 and CD73 expression on the surface of T cell subgroups 21 days after tumor induction were comparable in both models (Figure 6H,I). The majority of CD4^+^ and CD8^+^ T cells remained CD39 and CD73 negative.

### 2.4. The Immunogenic Composition of Inguinal Lymph Nodes Is Identical in Both Tumor Models and Is Not Affected by Tumor Formation

**Figure 7:**
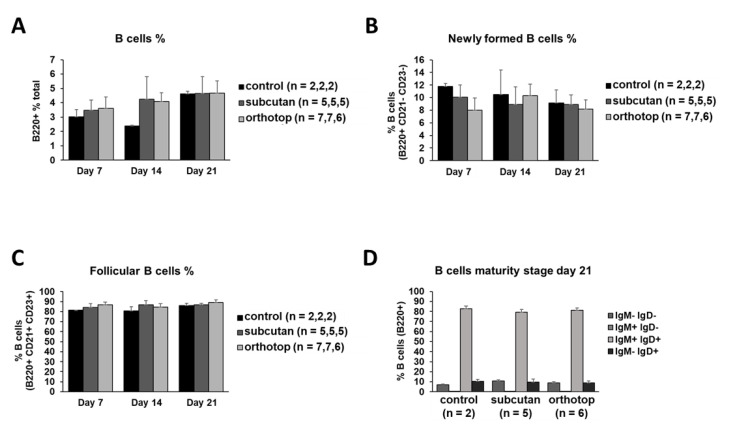

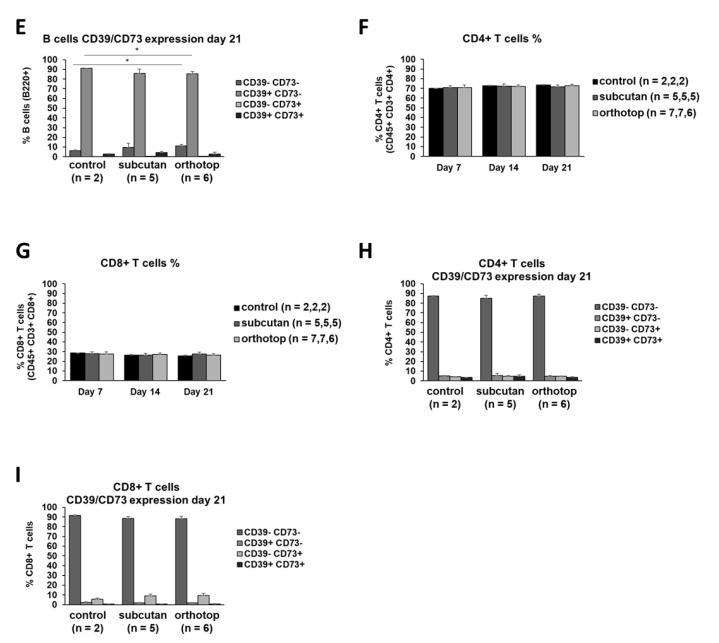
Cell populations in inguinal lymph nodes of orthotopic and subcutaneous HNSCC-bearing mice. (**A**–**E**) Populations of B cells and subgroups of B cells were analyzed using flow cytometry. (**D**,**E**) The expression profile for IgM/IgD and CD39/CD73 was determined in B cells. (**F**,**G**) Populations of CD4^+^ and CD8^+^ T cells were analyzed using flow cytometry. (**H**,**I**) The expression profile for CD39/CD73 was determined on T cells. Percentages were calculated based on relative lymphocyte numbers. Values are shown for day 0 (control), day 7, day 14, and day 21. On day 7, day 14, and day 21, the test group size for the subcutaneous group is *n* = 5, while for the control group *n* = 2. The test group size for the orthotopic group on day 7 is *n* = 7, day 14 *n* = 7, and day 21 *n* = 6. *p*-values < 0.05 were considered to be significant with (*). Data shown here are listed in Table S4.

Since the inguinal lymph nodes are much closer to the subcutaneously grown tumor than to the orthotopic grown tumor, we expected some variances in the immunogenic composition during tumor formation. In contrast to that local proximity, the relative amount of all B cells as well as in the B cell subpopulations remained equal over time in all experimental settings (Figure 7A–C). There were no detectable differences in the IgM and IgD expression on B cells in lymph nodes (Figure 7D). The CD39 and CD73 expression on B cells revealed some minor differences between the control group and the orthotopic tumor group (Figure 7E). Moreover, the relative amount of T cells, as well as the CD39 and CD73 expression on T cells, was shown to be equal in all experimental groups (Figure 7F–I). As in the spleen, the majority of T cells (nearly 90%) in lymph nodes was shown to be CD39- and CD73-negative (Figure 7H,I).

### 2.5. Tumor Formation Leads to Higher B Cell Proliferation and B Cell Mobilization in Bone Marrow

**Figure 8:**
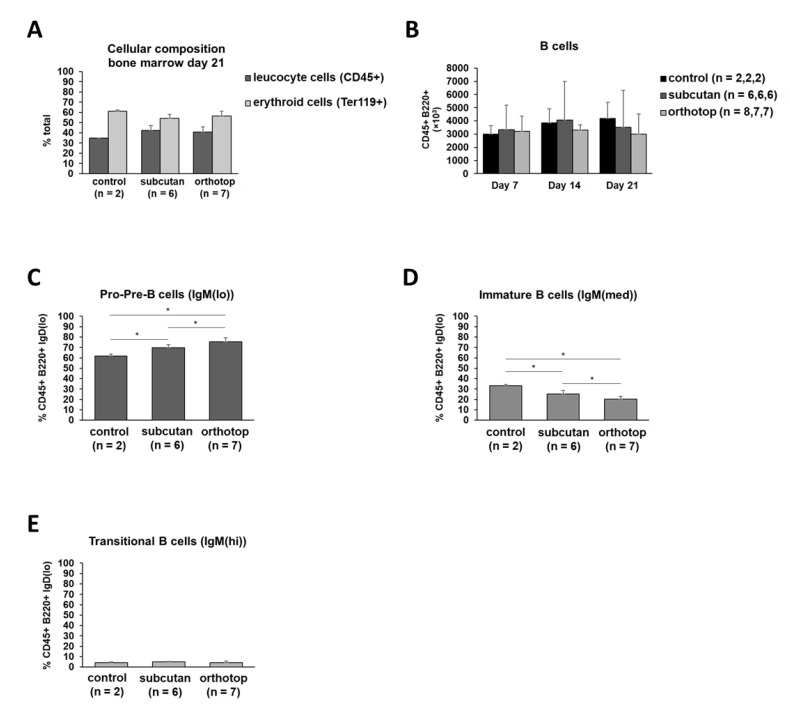
Cellular composition and B cell characterization in the bone marrow of orthotopic and subcutaneous HNSCC-bearing mice. (**A**) Cells of the bone marrow were harvested and divided into the erythroid and lymphoid cell lineage using flow cytometry. (**B**) B cells in the bone marrow on day 7, day 14, and day 21 after tumor induction are compared to healthy control mice. (**C**–**E**) Early B cell forms (IgD(lo)) are characterized by IgM expression intensity. On day 7, day 14, and day 21 the test group size for the subcutaneous group is *n* = 6, while for the control group *n* = 2. The test group size for the orthotopic group on day 7 is *n* = 8, day 14 *n* = 7, and day 21 *n* = 7. *p*-values < 0.05 were considered to be significant with (*). Data shown here are listed in Table S5.

For a complete investigation of the cellular composition in the immunogenic microenvironment of various lymphoid organs of tumor-bearing mice, bone marrow has been analyzed. While the percentages of leukocyte cells and erythroid cells were comparable in the tumor-bearing mice and the healthy control mice (Figure 8A), we were able to detect some alterations in the relative amount of specific B cell subpopulations. Even though the absolute number of B cells did not show any differences (Figure 8B), the percentage of early pro- and pre-B cells was increased in tumor-bearing mice (Figure 8C). Furthermore, the orthotopic model revealed a significantly higher amount of those mentioned early B cells in comparison to the subcutaneous model (75% vs. 69%, *p* ≤ 0.024). Moreover, the percentage of B cells at a later stage of development (immature B cells) was significantly decreased in tumor-bearing mice (*p* ≤ 0.022, Figure 8D). Moreover, this observation was more pronounced in the orthotopic model compared to the subcutaneous one. Combining these findings with the constant number of transitional B cells in both tumor models (Figure 8E), these data suggest an increase in B cell proliferation and B cell mobilization in the bone marrow of tumor-bearing mice compared to healthy control mice. In addition, we were able to detect a significantly higher increase in B cell proliferation and B cell mobilization in the bone marrow of orthotopic tumor-bearing mice compared to mice bearing a subcutaneous tumor.

The absolute numbers of pro- and pre-B cells, immature B cells, and transitional B cells did not show any significant differences (data not shown). About 10% of the B cells in the bone marrow express IgD and are, therefore, classified as recirculating B cells (data not shown). The population of recirculating B cells did not show changes during tumor formation (data not shown). Nevertheless, these findings also strengthen earlier-described observations in this study showing a high number of B cells, particularly of newly formed B cells that left the bone marrow and entered the peripheral lymphoid organs, in the tumor microenvironment (TME) (Figure 3A–D). Taken together, we have demonstrated a systemic antitumoral reaction based on B cell proliferation and migration that starts in the bone marrow and finally enters the tumor tissue.

### 2.6. Tumor Formation and Location Has No Effect on B Cell and T Cell Populations in the Thymus

**Figure 9:**
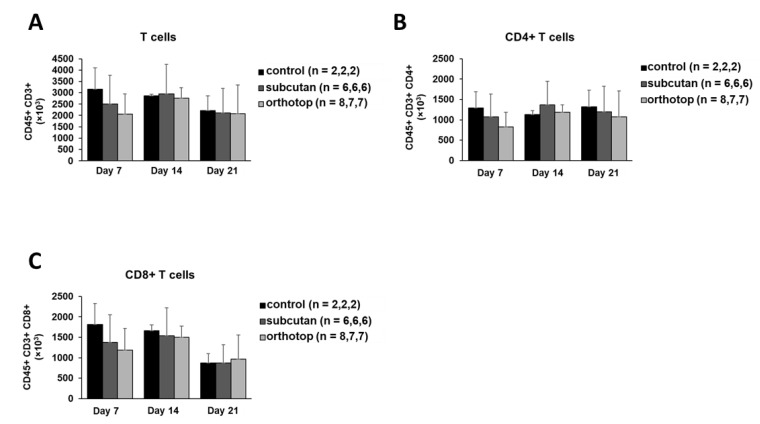
Cellular composition in the thymus of orthotopic and subcutaneous HNSCC-bearing mice. (**A**–**C**) Populations of T cells were analyzed using flow cytometry 7 days, 14 days, and 21 days after tumor induction. On day 7, day 14, and day 21 the test group size for the subcutaneous group is *n* = 6, while for the control group *n* = 2. The test group size for the orthotopic group on day 7 is *n* = 8, day 14 *n* = 7, and day 21 *n* = 7. Data shown here are listed in Table S6.

T cells pass through various development stages in the thymus before they migrate to their target regions. In these early stages, negative and positive selection takes place, which guarantees the correct formation of the T cell receptor. In our experiments, tumor formation and tumor location did not affect the analyzed cell populations in the thymus. The number of T cell subsets remained constant in the entire observed period (Figure 9A–C).

## 3. Discussion

### 3.1. The SCC VII-C3H/HeN System Is a Reliable Model to Study HNSCC in Immunocompetent Mice

In our experiments, we were able to induce a tumor with a volume of approximately 1 cm^3^ within 21 days. The implantation of 500,000 SCC VII cells led to a reliable tumor formation in the orthotopic model as well as in the subcutaneous model. Thus, the results confirm the dose used in previous studies to induce SCC VII tumors in C3H/HeN mice [11]. Subcutaneous tumor growth was largely unproblematic, while orthotopic tumor growth was occasionally accompanied by complications that led to the discontinuation of the experiment in rare cases (2/24 cases in total). Those complications were primarily caused by tumor growth in the direction of internal structures such as the esophagus and trachea. Consistent with previous studies [11], the bodyweight of tumor-bearing mice was the most reliable prognostic marker for the observation of the course of the disease.

### 3.2. A Larger Amount of B Cells, CD4^+^ T Cells, Macrophages, Dendritic Cells, and Granulocytes Creates a More Active TME in Orthotopic Grown Tumors, Accompanied by a Reduced Tumor Volume

In our studies, the subcutaneously grown tumor was significantly heavier than the orthotopically grown tumor 21 days after tumor induction. Although orthotopic tumors are smaller, they tend to have more TIL than the subcutaneous tumor, suggesting a more active tumor microenvironment. In reverse, the more active TME in orthotopic grown tumors could be the reason for the delayed tumor formation and consequently reduced tumor weight.

The immunogenic microenvironment is an image of the cellular and non-cellular components in the vicinity of the tumor cells. This composition determines the effectiveness of an immune reaction. The orthotopically grown tumor showed an increased number of B cells (1200 cells/g tumor) 21 days after tumor induction compared to the subcutaneous model (190 cells/g tumor). Most likely, the increase in B cell number is caused by the immigration of newly formed B cells generated in the bone marrow. Around 30% of tumor-infiltrating B cells co-express CD39 and CD73 21 days after tumor induction in both tumor models. Moreover, in the orthotopic tumor model, tumor-infiltrating B cells showed a significant gain of CD39/CD73 expression during tumor formation over time, while the number of CD39^−^ CD73^−^ B cells is reduced (Figure S1). Although the same tendency was observed in subcutaneous-induced tumors, this observation was not significant in this experimental subgroup.

Since the CD39 and CD73 expression, and therefore, the adenosine production, is linked to supporting an immunosuppressive microenvironment [18], the orthotopic microenvironment could be more pro-tumoral caused by an increased number of B cells expressing CD39 and CD73. In contrast, we found a reduced volume of orthotopic-induced tumors. Accordingly, the consequences of the overall increase in B cell numbers has to be further investigated. The increased population of B cells consists of several B cell subpopulations in a different activation status and, therefore, a different anti-tumoral potential. We observed a B cell subset of newly formed B cells to be significantly increased in the orthotopic model (Figure 3D). Despite the comparable IgM and IgD expression in both models, the newly formed B cells could represent a different state of activation among the tumor-infiltrating B cells.

Naive B cells entering the TME receive growth and differentiation signals from other TIL, such as T-helper cells, and compete for antigens presented by dendritic cells in a process known as affinity maturation [19]. At this point, the relevance of this maturation process gains importance because the population of CD4^+^ T cells and dendritic cells are significantly larger in the TME of the orthotopic system than in the subcutaneous system (Figure 2H and Figure 4A). Ultimately, those B cells can aggregate with other immune cells in non-lymphoid tissues forming tertiary lymphoid structures [20]. Furthermore, we were able to trace the anti-tumoral B cell reaction back to the bone marrow. Representing the origin of lymphoid cells, we detected an increased population of pro- and pre-B cells in both tumor models (Figure 8C). Additionally, this increase in early B cell precursors was significantly higher in the orthotopic tumor model suggesting a systemic reaction towards tumor formation largely represented by B cell formation. Therefore, a direct influence of increased B cell population on tumor formation can be suggested.

However, a contribution of other immune cell populations, such as T cells or myeloid cells, is more likely to contribute to the formation of an anti-tumoral TME in the orthotopic model, and therefore, should be considered here [19,21]. In support of this assumption, we detected an increased number of CD4^+^ T cells in the orthotopic tumor, while the number of CD8^+^ T cells was comparable between both tumor localizations. A higher number of tumor-infiltrating T cells has been considered as a sign of better prognosis in head and neck cancer [22,23,24,25]. Our data are consistent with previous results, which also describe an increased CD4^+^ T cell population in the orthotopic tumor [11]. An increased number of CD4^+^ T cells was correlated with both better locoregional control and longer survival of HNSCC patients [23]. Interestingly, within the CD4^+^ T cell population, we identified 30% of CD39^+^CD73^+^ regulatory T cells (Treg) in the TME of subcutaneously grown tumors. In contrast, we observed only 15% of CD39^+^CD73^+^ regulatory T cells in the TME of orthotopically grown tumors. An increased accumulation of Tregs was reported in the tumor site and peripheral blood of patients with cancer, including HNSCC, compared to healthy controls [26]. Since Treg are associated with reduced anti-tumoral immune responses against HNSCC [23,27], our results indicate an attenuation of the immunosuppressive milieu in the orthotopic model. This attenuation is not only caused by decreased numbers of Treg but also by a generally increased number of B cells and CD4^+^ T lymphocytes. Ultimately, the role of Treg cells in HNSCC remains unclear and has yet to be further clarified.

The number of myeloid cells in both tumors increased during tumor growth and was comparable 21 days after tumor induction regardless of the tumor location. However, only in the orthotopic model, the increase in granulocytes, macrophages, and dendritic cells reached significant levels during tumor formation. In line with a previous study [11], we found the absolute granulocyte numbers within the TME of the orthotopic-induced tumor slightly reduced in comparison to the subcutaneous tumor model (Figure 2F). In summary, the decreased volume of the orthotopic grown tumor can be explained by a change in the cellular microenvironment caused by a different tumor induction site. This change in immune cell composition eventually leads to a more anti-tumoral microenvironment.

### 3.3. An Increase in the White Blood Cell Count in Peripheral Blood Indicates a Systemic Inflammatory Reaction

Towards the end of the test period, an increased blood leukocyte count was observed. The population of predominantly CD39/CD73 negative B and T lymphocytes in the blood remained unchanged by tumor growth. The increase of leukocytes in the blood of tumor-bearing mice was already demonstrated in other studies [28]. This behavior is likely to be interpreted as a systemic inflammatory response to the tumor. For the immune system, tumors are “wounds that do not heal” [29]. These “wounds” cause a corresponding increase of inflammatory parameters such as leukocytes. The small number of regulatory B cells in the blood of tumor-bearing mice was already described to be in contrast to the situation in HNSCC patients, where over 60% of blood B cells express CD39 and CD73 [9]. This finding can be linked to the pathogen free living conditions of mice, and therefore, to the reduced activation of the immune system in which less regulatory B cells are needed to prevent an overreaction of the immune system [30].

### 3.4. B Cell and T Cell Populations in Spleen, Lymph Nodes, and Thymus Were Not Affected by Tumor Induction

The composition of immune cell subsets in peripheral lymphoid organs, such as the spleen, inguinal lymph nodes, and thymus, was not altered during tumor formation. An inflammatory response against tumor cells in the lymph nodes would be led primarily by NK cells, against which SCC VII cells show reduced vulnerability [31,32]. However, NK cells were not characterized in the conducted analysis. An inflammatory reaction in the lymph nodes was not detected. In previous research, when establishing a model of lymph node metastasis using SCC VII cells, a change in their morphology was present. In histological examinations, an increased number of germinal centers was observed [33]. However, the experimental design differs markedly from the design applied here. In order to generate lymph node metastasis, 1 × 10^6^ SCC VII cells were injected intramuscularly into the musculus gastrocnemius. Thus, in our studies, a systemic anti-tumor reaction affecting immune cell composition and function outside the tumor could not be observed when the tumor was induced subcutaneously or orthotopically. However, follicular structures and germinal center formation have not been analyzed here.

### 3.5. The SCC VII-C3H/HeN Mouse Model Reflects a Situation Comparable to HNSCC Patients

Besides neoplastic cells, the tumor microenvironment contains a significant amount of non-cancerous stromal cells. These includes fibroblasts, myeloid-derived cells such as macrophages and dendritic cells, and of course effector cells such as T cells and B cells. Those populations play an important role in disease outcome by forming the tumor microenvironment. CD8^+^ and CD4^+^ T cells are essential for tumor containment [34]. As mentioned above, the infiltration of T cells into the tumor tissue represents a prognostic marker for HNSCC patients. A decreased absolute count of T lymphocytes was observed in the blood of patients suffering from HNSCC [35]. Therefore, by tracing the cellular subgroups of the immune system in various organs in our murine HNSCC model, we were able to reproduce observations in human patients. Since a shift in pro-apoptotic and anti-apoptotic proteins, as well as molecular defects in circulating CD8^+^ T cells in the blood of HNSCC patients, have been observed [36], molecular characterization of effector cell subgroups could be performed in the SCC VII/C3H/HeN mouse model in a more controlled environment.

Effector cells of innate immunity, such as NK cells, dendritic cells, granulocytes, or macrophages, have already been a target of intensive research in HNSCC treatment. HNSCC cells successfully inhibit NK cell function [37,38]. In this regard, an extended application of the mouse model, shown here, for example, by an additional extraction and analysis of NK cells, is possible. An increased amount of dendritic cells in the tumor tissue of patients is associated with longer disease-free survival [39], whereas fewer dendritic cells are found in high-grade tumors [40]. In the orthotopic tumor model, we were able to detect an increase in the number of dendritic cells (Figure 2H) during tumor formation. This observation can be caused by the localization of tumor growth. The mucosa of the aerodigestive tract, in which a high number of dendritic cells can be expected [41], is connected to the orthotopic specimen. This local proximity is not present in the subcutaneous model, and therefore, a higher number of dendritic cells cannot be expected in this model. In addition to dendritic cells, HNSCC tumor formation leads to an accumulation of other myeloid cells, including macrophages and granulocytes in patients due to the inflammatory process described above. Dendritic cells, as well as macrophages, highly interact with other immune cell populations, such as T cells and B cells. In addition to their interaction with other immune cell populations, tumor-associated macrophages (TAMs) influence the development of a tumor by secreting proteases and vascular endothelial growth factor (VEGF), causing a higher microvessel density (compare Figure 1(A2,B2)) [38,42,43]. To target these observations in HNSCC patients, the mouse model demonstrated here can provide a reliable tool for the further investigation of the interconnected relationships between cell populations in TME.

Moreover, an increase in the number of Treg cells was either proposed to be a positive [23,44] or a negative prognostic marker in HNSCC [45,46]. A retrospective study of HNSCC patients found that the localization of the protein FOXP3 in the cytoplasm of CD4^+^ T cells correlated with a favorable prognosis or strongly predicted recurrence. In this context, the SCC VII-C3H/HeN mouse model can provide all cellular components for further exploration of this observation since CD4^+^ T cells and Treg cells are easily identified within the TME (Figure 4A,C,D).

As a most striking characteristic, the B cell enrichment and increased B cell development in orthotopic grown tumors could be revealed in the mouse model described here. Besides the generation of tumor antigen antibodies, the role of B cells in the TME of solid tumors has to be further characterized. In addition to the adaptive immune defense, B cells have been described to process antigens and acquire immunosuppressive properties. Until today, it remains unclear whether B cells have a pro- or anti-tumoral role during tumor formation. On the one hand, B cells have been shown to suppress T cells and promote tumor growth [47,48]. On the other hand, tumor-infiltrating B cells are associated with a favorable outcome in patients with HNSCC [45]. Previously, we have described the role of an immunosuppressive B cell subpopulation in HNSCC patients [9]. In this study, we focused on regulatory B cells (Breg), which promote tumor formation by producing adenosine via CD39 and CD73. By using the SCC VII-C3H/HeN mouse model, we were able to detect the population of adenosine producing Breg we observed in the blood and tumor tissue of HNSCC patients within a living model, and thus, further elucidated the role of Breg in the immune system. In summary, the work described here shows that the SCC VII-C3H/HeN system is applicable in proving newly established immunotherapeutic interventions.

## 4. Materials and Methods

### 4.1. Cell Culture

SCC VII cells were maintained in complete Roswell Park Memorial Institute (RPMI) 1640 medium (Gibco, Carlsbad, CA, USA), containing 10% heat-inactivated fetal bovine serum (FBS, Biochrom, Berlin, Germany), 100 U/mL penicillin, and 100 mg/mL streptomycin (PAN Biotech, Aidenbach, Germany) and were cultured in 37 °C and 5% CO_2_. To prepare SCC VII cells for injection, cultured cells were trypsinized and washed twice with Dulbecco‘s-Phosphate-Buffered-Saline (DPBS) Medium (Gibco, Carlsbad, CA, USA).

### 4.2. Animal Model

Five- to six-week-old male C3H/HeN mice were obtained from Janvier Labs (Le Genest-Saint-Isle, France). The animals were housed in groups of five in standard housing conditions in sterilized plastic cages in a temperature-controlled room with a 12-h dark-light cycle and received tap water and forage ad libitum. After at least two weeks of accommodation, blood was drawn from eight mice (two predefined control mice and three mice from each experimental group) to guarantee a reliable starting point. After that, 5 × 10^5^ murine squamous carcinoma cells (SCC VII) in a total volume of 50 µL DPBS were injected into the floor of the mouth of syngeneic mice to induce orthotopic tumor formation as described earlier [11]. For subcutaneous tumor growth, 5 × 10^5^ murine squamous carcinoma cells (SCC VII), in a total volume of 200 µL DPBS, were injected into the right flank of syngeneic mice. Before tumor induction (day 0) and on day 7, day 14, and day 21 after tumor injection, blood was taken (facial vein) and analyzed by an animal blood counter (Scil animal care company GmbH, Viernheim, Germany) and flow cytometry. Mice were weighed daily, and the overall health condition was evaluated by a specially designed scoring system. Mice were euthanized after weight loss exceeding 20%, or if they displayed tumor-related morbidity. Experiments were terminated 21 days after tumor inoculation. On day 21, the mice were sacrificed, and the tumor as well as lymphoid organs were harvested and processed as described below. The tumor was weighed and tumor-infiltrating lymphocytes (TIL) were isolated and analyzed by flow cytometry. Cells from the spleen, lymph nodes, thymus, and bone marrow were processed and analyzed by flow cytometry with the GALLIOS Flow Cytometer (Beckman Coulter, Brea, CA, USA). Animal experiments were performed with the approval of the regional animal ethics committee, Regierungspräsidium Tübingen, Germany (Protocol Number #1270).

A total of 48 mice were divided into a control group containing 6 mice, an experimental group with subcutaneous tumor induction (18 mice), and a second experimental group with orthotopic tumor induction (24 mice). On day 7, day 14, and day 21, a total of 16 mice (8 with an orthotopic tumor, 6 with a subcutaneously grown tumor, and 2 control mice) were removed and analyzed as described below. The tumor volume on day 7 was insufficient (tumor diameter smaller than 3 mm) for further lymphocyte extraction in both experimental groups. Two mice bearing an orthotopic tumor were removed from the experiment before reaching day 14 and day 21 due to adverse events. After 14 and 21 days, one orthotopic tumor sample was excluded from the analyses since it was too small for any further preparation. One orthotopic lymph node sample and a subcutaneous lymph node sample had to be excluded from further analyses because no cells could be extracted. On day 21, one spleen sample of a subcutaneous tumor-bearing mouse and two blood samples of mice bearing an orthotopic tumor had to be excluded from further analyses due to incorrect antibody staining.

### 4.3. Tumor Preparation

After extraction, the tumor was weighed and cut into small pieces. Minced tissue pieces were collected in RPMI containing 200 IU/mL collagenase I (Pan-Biotech, Aidenbach, German) for at least two hours at 37 °C. After digestion, the tissue was mashed with a 100 µm EASY strainer (Greiner Bio-One, Frickenhausen, Germany). The lymphocyte fraction was isolated via Biocoll centrifugation. After processing, the number of vital cells was determined manually by Neubauer chamber counting.

### 4.4. Spleen, Lymph Node, and Thymus Preparation

After extraction, the organ was mashed with a 40 µm EASY strainer (Greiner Bio-One, Frickenhausen, Germany) and the separated cells were counted manually using the Neubauer chamber counting method.

### 4.5. Antibodies and Reagents

The following anti-mouse monoclonal Antibodies (mAbs) were used for flow cytometry: CD25 APC, IgM APC, CD3e Pacific Blue, CD11b Pacific Blue, CD3 FITC, CD8a FITC, IgD FITC, MHC-II FITC, CD39 PE-Cyanin7 (all eBioscience, Waltham, MA, USA); CD4 APC/Cy7, CD21/CD35 APC/Cy7, IgD APC/Cy7, CD73 Pacific Blue, CD8a PE, B220/CD45R PE-Cyanin7, CD19 PE-Cyanin7, F4/80 PE-Cyanin7, Ly6G PerCP/Cy5.5 (all BioLegend, San Diego, CA, USA); CD11c APC, CD45 APC/Cy7, B220/CD45R FITC, CD23 PE, CD44 PE, CD4 PerCP (all BD Biosciences, San Jose, CA, USA); IgM PE (all SouthernBiotech, Birmingham, AL, USA); Ly6C PE, Ter119 PE (all Miltenyi Biotech, Bergisch Gladbach, Germany).

### 4.6. Surface Staining

A maximum of 1 × 10^6^ cells were incubated with mAbs at RT for 20 minutes in the dark, washed, and collected in PBS with 0.5% BSA for flow cytometry analysis. Flow cytometry was performed using Gallios 10-color-flow-cytometer equipped with Kaluza flow cytometry software version 1.3 (Beckman Coulter, Brea, CA, USA).

### 4.7. Statistical Analysis

All data are presented as means ± standard deviation (SD). Error bars indicate the SD of the mean. Data were analyzed for their distribution by the Shapiro-Wilk normality test. All data sets mentioned here are normally distributed. The two-sided student’s *t*-test was used for the nonparametric analysis of two mean values. *p*-values ≤ 0.05 were considered to be significant. Statistical analysis was performed using the program Excel version 2019 (Microsoft, Redmond, WA, USA) as well as GraphPad Prism version 8 (GraphPad Software, San Diego, CA, USA).

## 5. Conclusions

In summary, our study demonstrated numerous differences between subcutaneous and orthotopic HNSCC models. Since the aim of an animal model is to represent the situation in humans as close as possible, the localization of the tumor and the composition of the immunogenic microenvironment are important factors that need to be accounted before establishing a tumor model. While the immunogenic microenvironments in the spleen, lymph nodes and thymus are equal in both HNSCC models, a systemic immune reaction can be detected in the bone marrow and blood of tumor-bearing mice. Although these systemic reactions are only slightly affected by the tumor location, the most relevant differences can be shown directly in the immunogenic microenvironment of the tumor. There, the orthotopic model contained more B cells and CD4^+^ T cells than the subcutaneous model. Since this altered immunogenic tumor microenvironment may also be associated with an altered response to treatment attempts, interventional studies of a subcutaneously grown tumor most likely lead to different results compared to treatment of an orthotopic grown tumor. Furthermore, we visualized an important role of B cells during tumor formation. Despite the more difficult handling properties of an orthotopic HNSCC model, the orthotopic model provides a more physiologic representation of tumors in the head and neck region, and therefore, a comparable situation to HNSCC patients. Altogether, our investigation suggests that the orthotopic tumor model is superior to the subcutaneous tumor model for further research with therapeutic approaches.

## Data Availability

The data that support the findings of this study are available from the corresponding author upon reasonable request.

## Supplementary Materials

The following are available online at https://www.mdpi.com/1422-0067/22/1/247/s1.

## Abbreviations

ATP	adenosine-triphosphate
Breg	regulatory B cells
CD	cluster of differentiation
DOAJ	Directory of open access journals
FOXP3	forkhead-box-protein P3
HNC	head and neck cancer
HNSCC	head and neck squamous cell carcinoma
HPV	human papillomavirus
mAb	monoclonal antibody
MDPI	Multidisciplinary Digital Publishing Institute
NK cells	natural-killer cells
SD	standard deviation
TAM	tumor associated macrophages
TIL	tumor-infiltrating lymphocytes
TLR-4	toll-like-receptor-4
TME	tumor micoenvironment
Treg	regulatory T cells
VEGF	vascular endothelial growth factor
WBC	white blood cell count
