# Supplementary material for: Characterization and Differentiation of the Tumor Microenvironment (TME) of Orthotopic and Subcutaneously Grown Head and Neck Squamous Cell Carcinoma (HNSCC) in Immunocompetent Mice
Source: Int J Mol Sci. 2020 Dec 29;22(1):247. doi: 10.3390/ijms22010247 (PMC7796118; doi:10.3390/ijms22010247)
Supplement: Supplementary file 1 [file ijms-22-00247-s001.pdf]

Characterization and Differentiation of the Tumor Microenvironment (TME) of Orthotopic and Subcutaneously Grown Head and Neck Squamous Cell Carcinoma (HNSCC) in Immunocompetent Mice

Matthias Brand, Simon Laban, Marie-Nicole Theodoraki, Johannes Doescher, Thomas K. Hoffmann, Patrick J. Schuler and Cornelia Brunner

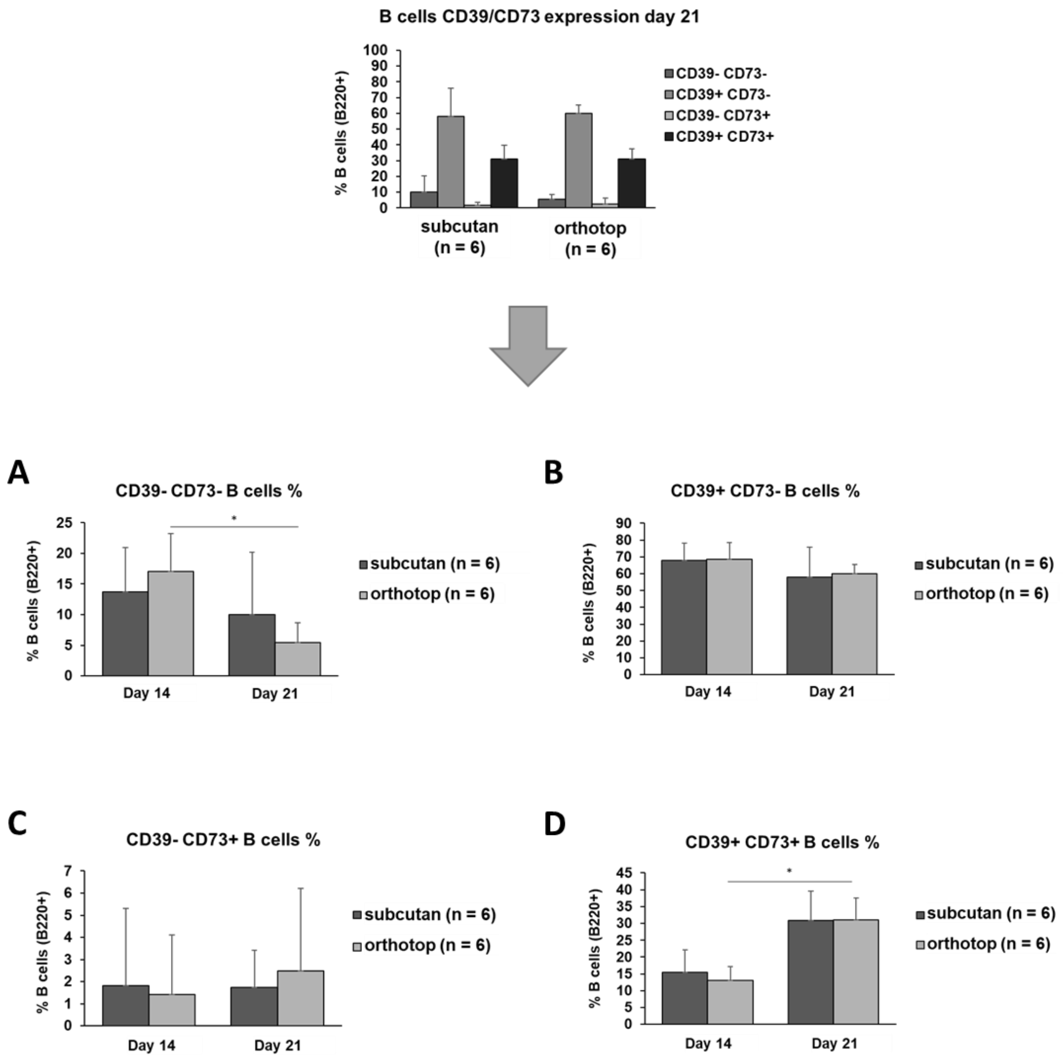

Figure S1. CD39/CD73 expression on B cells in the tumor of orthotopic and subcutaneous HNSCC bearing mice. 14 days and 21 days after tumor induction, tumors were harvested and populations of B cells with a different CD39/CD73 expression were analyzed using flow cytometry. On day 14 and day 21 the test group size for the subcutaneous group is $n = 6$, for the orthotopic group $n = 6$. p-values < 0.05 were considered to be significant with (*).

Table S1. Tumor-data summary of orthotopic and subcutaneous HNSCC bearing mice. Data is visualized in Figure 2–4. SD = standard deviation.

		Subcutan				Orthotop			
		Day 14		Day 21		Day 14		Day 21	
		**Mean**	SD	**Mean**	SD	**Mean**	SD	**Mean**	SD
	Tumorweight (g)	**0.36**	0.13	**0.75**	0.22	**0.31**	0.12	**0.47**	0.12
	TIL (×10³)	**19,958.33**	15,422.13	**48,960.00**	30,610.41	**10,691.67**	7617.71	**36,800.00**	9025.52
	TIL/tumorweight (×10³/g)	**55,696.13**	39,132.27	**59,633.87**	23,554.93	**31,143.58**	10,735.44	**81,098.27**	34366.33
% Myeloid cells	Macrophages			**60.11**	3.30			**60.65**	3.41
	Granulocytes			**12.03**	2.15			**16.23**	5.98
	Monocytes			**3.62**	0.74			**2.59**	0.87
	Dendritic cells			**24.69**	2.36			**22.79**	1.53
	Macrophages (×10³)	**1077.85**	608.75	**2595.01**	1366.30	**745.69**	446.25	**1731.31**	769.28
	Granulocytes (×10³)	**288.77**	112.96	**532.61**	314.05	**201.85**	116.07	**452.58**	170.15
	Monocytes (×10³)	**151.08**	101.81	**173.59**	134.65	**40.36**	15.82	**71.56**	30.32
	Dendritic cells (×10³)	**514.04**	293.24	**1064.87**	596.93	**308.72**	173.29	**647.32**	274.00
	B cells (%)	**0.77**	0.45	**0.34**	0.26	**1.93**	1.76	**1.58**	0.59
	B cells (×10³)	**155.67**	137.29	**177.41**	157.00	**125.68**	96.47	**541.82**	153.54
	B cells/tumorweight (×10³/g)	**449.87**	443.68	**194.09**	150.10	**488.64**	419.55	**1206.85**	503.18
	Newly formed B cells (×10³)	**93.17**	81.57	**56.24**	38.43	**76.42**	53.64	**305.83**	97.35
	Follicular B cells (×10³)	**44.25**	38.46	**108.45**	163.68	**54.41**	33.14	**128.99**	83.88
% B cells	IgM- IgD-			**10.91**	4.06			**9.17**	2.68
	IgM+ IgD-			**1.21**	1.01			**0.98**	0.35
	IgM+ IgD+			**84.48**	3.68			**85.66**	3.85
	IgM- IgD+			**0.83**	1.13			**3.62**	4.25
	CD39- CD73-			**10.02**	10.21			**5.40**	3.29
	CD39+ CD73-			**58.02**	17.84			**59.97**	5.41
	CD39- CD73+			**1.75**	1.67			**2.47**	3.75
	CD39+ CD73+			**30.84**	8.73			**31.03**	6.45
	CD4+ T cells (×10³)	**292.24**	211.43	**299.84**	121.40	**275.63**	101.02	**540.94**	72.96
	CD8+ T cells (×10³)	**52.12**	45.33	**58.42**	36.49	**34.65**	23.40	**63.83**	24.09
	Regulatory T cells (% CD4+ T cells)	**28.49**	4.56	**29.90**	4.75	**16.02**	5.71	**16.77**	7.14
	Regulatory T cells (×10³)	**94.79**	64.88	**61.88**	33.02	**28.44**	15.52	**52.48**	17.84
% CD4+ T cells	CD39- CD73-			**17.36**	5.57			**37.10**	15.31
	CD39+ CD73-			**17.03**	6.87			**12.36**	7.46
	CD39- CD73+			**17.34**	6.65			**19.56**	4.43
	CD39+ CD73+			**48.27**	5.70			**30.98**	10.59
% CD8+ T cells	CD39- CD73-			**71.63**	8.09			**70.76**	5.97
	CD39+ CD73-			**5.73**	2.06			**4.57**	1.61
	CD39- CD73+			**18.15**	4.72			**18.42**	3.56
	CD39+ CD73+			**4.50**	2.37			**6.25**	1.78

Table S2. Data summary of peripheral blood of orthotopic and subcutaneous HNSCC bearing mice. Data is visualized in Figure 5. SD = standard deviation.

		Control								Subcutan						Orthotop					
		Day 0		Day 7		Day 14		Day 21		Day 7		Day 14		Day 21		Day 7		Day 14		Day 21	
		**Mean**	SD	**Mean**	SD	**Mean**	SD	**Mean**	SD	**Mean**	SD	**Mean**	SD	**Mean**	SD	**Mean**	SD	**Mean**	SD	**Mean**	SD
	WBC ($\times 10^3$)	**8.59**	0.90	**10.27**	0.03	**7.64**	0.23	**10.38**	0.38	**9.81**	0.35	**11.15**	1.76	**14.38**	1.40	**10.24**	0.79	**11.57**	0.62	**13.56**	1.39
	Lymphocytes ($\times 10^3$)	**5.77**	0.77	**7.60**	0.60	**5.65**	0.25	**7.15**	0.55	**7.02**	0.25	**6.70**	0.45	**8.40**	1.36	**6.98**	0.43	**7.17**	0.68	**7.60**	1.03
	B cells/μL	**279.57**	62.28	**293.86**	18.92	**157.60**	36.29	**339.99**	10.52	**371.07**	144.45	**492.26**	57.28	**357.55**	193.34	**362.97**	183.54	**334.41**	110.88	**476.76**	203.03
	T cells/μL	**1729.36**	602.02	**2914.21**	211.56	**1629.85**	76.59	**2726.95**	46.39	**3141.56**	575.74	**2025.83**	722.58	**2418.24**	867.25	**2466.38**	349.06	**2628.27**	425.25	**2314.46**	512.37
	CD4+ T cells/μL	**1264.88**	435.77	**2076.40**	159.14	**1129.02**	41.43	**1966.65**	48.17	**1869.87**	919.41	**1454.37**	528.37	**1725.61**	615.66	**1513.10**	625.34	**1890.21**	315.42	**1690.68**	375.58
	CD8+ T cells/μL	**416.86**	149.80	**766.45**	35.93	**465.76**	27.74	**713.04**	6.95	**677.54**	340.46	**520.07**	178.07	**612.99**	221.18	**574.67**	234.05	**646.18**	110.16	**572.84**	136.27
% B cells	IgM- IgD-							**3.51**	0.25					**3.19**	1.32					**3.83**	0.79
	IgM+ IgD-							**27.46**	2.87					**22.63**	5.07					**14.00**	2.70
	IgM+ IgD+							**56.55**	2.30					**55.06**	5.79					**47.73**	5.34
	IgM- IgD+							**13.92**	0.35					**19.36**	3.35					**34.56**	5.92
	CD39- CD73-							**8.34**	1.64					**11.99**	5.79					**10.65**	2.18
	CD39+ CD73-							**88.90**	2.26					**85.93**	5.56					**86.87**	1.80
	CD39- CD73+							**0.02**	0.02					**0.01**	0.01					**0.04**	0.04
	CD39+ CD73+							**1.58**	0.35					**1.42**	0.40					**1.88**	0.95
% CD4+ T cells	CD39- CD73-							**84.74**	2.29					**81.87**	1.67					**82.31**	2.78
	CD39+ CD73-							**4.89**	0.28					**6.71**	1.23					**5.82**	1.05
	CD39- CD73+							**6.67**	1.55					**7.08**	1.07					**7.58**	1.04
	CD39+ CD73+							**3.71**	0.47					**4.34**	0.66					**4.30**	1.14
% CD8+ T cells	CD39- CD73-							**82.78**	0.29					**81.10**	2.11					**79.39**	2.47
	CD39+ CD73-							**4.96**	1.82					**3.31**	1.00					**3.02**	0.94
	CD39- CD73+							**11.25**	1.94					**14.26**	2.49					**16.33**	1.89
	CD39+ CD73+							**1.03**	0.18					**1.33**	0.33					**1.26**	0.21

Table S3. Data summary of the spleen of orthotopic and subcutaneous HNSCC bearing mice. Data is visualized in Figure 6. SD = standard deviation.

		Control						Subcutan						Orthotop					
		Day 7		Day 14		Day 21		Day 7		Day 14		Day 21		Day 7		Day 14		Day 21	
		**Mean**	SD	**Mean**	SD	**Mean**	SD	**Mean**	SD	**Mean**	SD	**Mean**	SD	**Mean**	SD	**Mean**	SD	**Mean**	SD
B cells (×10³)		**3622.81**	1062.30	**5575.03**	543.24	**4697.77**	12.66	**4243.63**	2032.28	**6166.90**	2559.29	**7375.98**	1163.84	**3416.95**	715.36	**5360.60**	1016.01	**7190.67**	3522.21
Newly formed B cells (×10³)		**192.08**	59.92	**295.57**	38.15	**233.51**	18.49	**287.89**	140.37	**326.67**	181.39	**487.90**	103.73	**254.52**	85.24	**356.87**	140.32	**386.12**	191.95
Follicular B cells (×10³)		**2336.99**	806.46	**3106.55**	666.32	**3678.11**	534.66	**3441.83**	1654.43	**3382.75**	1497.19	**5502.21**	1063.01	**2789.70**	725.97	**3540.29**	1297.84	**4874.28**	2067.85
% B cells	IgM- IgD-					**11.58**	2.30					**12.44**	3.16					**11.00**	1.33
	IgM+ IgD-					**13.34**	3.89					**34.40**	5.17					**25.25**	4.88
	IgM+ IgD+					**44.07**	7.79					**36.93**	7.20					**48.82**	6.72
	IgM- IgD+					**30.75**	1.51					**16.01**	1.36					**13.08**	2.53
	CD39- CD73-					**4.45**	0.47					**3.92**	1.32					**4.85**	1.78
	CD39+ CD73-					**88.05**	0.23					**88.50**	2.91					**89.33**	4.04
	CD39- CD73+					**0.00**	0.00					**0.01**	0.01					**0.01**	0.01
	CD39+ CD73+					**7.12**	0.29					**7.33**	2.47					**5.54**	2.89
CD4+ T cells (×10³)		**5976.48**	1389.25	**8087.21**	3772.45	**7797.68**	431.95	**6313.18**	3210.18	**7663.58**	3021.93	**6909.76**	1534.10	**4602.28**	1517.70	**7746.30**	2863.00	**7770.22**	1831.97
CD8+ T cells (×10³)		**2877.33**	684.99	**3766.17**	2222.86	**4199.72**	637.39	**2960.31**	1844.94	**3911.25**	1652.32	**3748.45**	1085.30	**2505.41**	863.03	**4015.26**	1724.62	**3997.91**	759.14
% CD4+ T cells	CD39- CD73-					**57.77**	11.51					**54.80**	3.43					**56.66**	6.38
	CD39+ CD73-					**18.07**	4.91					**25.14**	4.38					**17.03**	7.64
	CD39- CD73+					**4.58**	0.36					**5.13**	1.24					**8.75**	3.36
	CD39+ CD73+					**19.57**	6.96					**14.93**	3.80					**16.99**	3.07
% CD8+ T cells	CD39- CD73-					**80.88**	2.37					**74.87**	2.49					**75.08**	3.71
	CD39+ CD73-					**4.24**	0.79					**6.80**	1.11					**5.10**	3.02
	CD39- CD73+					**10.63**	0.13					**11.62**	1.27					**16.04**	4.09
	CD39+ CD73+					**4.25**	1.71					**6.91**	1.01					**5.07**	3.48

Table S4. Data summary of inguinal lymph nodes of orthotopic and subcutaneous HNSCC bearing mice. Data is visualized in Figure 7. SD = standard deviation.

		Control						Subcutan						Orthotop					
		Day 7		Day 14		Day 21		Day 7		Day 14		Day 21		Day 7		Day 14		Day 21	
		**Mean**	SD	**Mean**	SD	**Mean**	SD	**Mean**	SD	**Mean**	SD	**Mean**	SD	**Mean**	SD	**Mean**	SD	**Mean**	SD
B cells (% total)		**3.03**	0.49	**2.38**	0.04	**4.61**	0.20	**3.48**	0.73	**4.23**	1.58	**4.63**	1.19	**3.60**	0.80	**4.08**	0.62	**4.66**	0.86
Newly formed B cells (% B cells)		**11.79**	0.47	**10.51**	3.87	**9.16**	2.09	**10.07**	1.94	**8.91**	2.82	**8.94**	1.51	**8.00**	1.95	**10.33**	1.82	**8.16**	1.49
Follicular B cells (% B cells)		**81.52**	0.03	**80.84**	4.20	**85.90**	2.47	**84.08**	3.86	**86.86**	4.00	**86.89**	1.43	**86.82**	2.59	**84.51**	3.33	**89.25**	2.37
% B cells	IgM- IgD-					**7.05**	0.66					**10.76**	1.24					**9.03**	0.83
	IgM+ IgD-					**0.14**	0.07					**0.13**	0.05					**0.12**	0.09
	IgM+ IgD+					**82.57**	2.80					**79.21**	2.70					**81.40**	1.92
	IgM- IgD+					**10.35**	2.00					**9.73**	2.94					**8.81**	2.10
	CD39- CD73-					**6.26**	0.63					**9.59**	4.27					**12.22**	11.30
	CD39+ CD73-					**90.96**	0.35					**85.89**	4.41					**84.25**	85.44
	CD39- CD73+					**0.00**	0.00					**0.03**	0.02					**0.00**	0.01
	CD39+ CD73+					**2.90**	0.37					**4.35**	1.29					**3.26**	2.83
CD4+ T cells (% T cells)		**65.34**	0.27	**68.33**	0.26	**67.05**	1.08	**64.83**	2.65	**64.68**	3.31	**57.48**	3.11	**66.01**	3.52	**63.56**	2.05	**58.86**	3.76
CD8+ T cells (% T cells)		**26.34**	0.18	**26.20**	0.30	**24.20**	0.91	**26.30**	1.07	**23.76**	1.52	**22.95**	1.72	**25.49**	1.38	**24.37**	1.69	**21.62**	1.57
% CD4+ T cells	CD39- CD73-					**87.34**	0.32					**85.09**	3.07					**87.36**	1.87
	CD39+ CD73-					**4.89**	0.63					**5.61**	2.18					**4.51**	0.84
	CD39- CD73+					**4.17**	0.27					**4.49**	0.86					**4.51**	0.71
	CD39+ CD73+					**3.60**	0.05					**4.81**	1.34					**3.62**	0.68
% CD8+ T cells	CD39- CD73-					**91.55**	0.56					**88.73**	1.56					**88.30**	2.28
	CD39+ CD73-					**2.26**	0.47					**1.78**	0.50					**1.81**	0.23
	CD39- CD73+					**5.70**	1.00					**8.82**	1.90					**9.26**	2.26
	CD39+ CD73+					**0.49**	0.04					**0.67**	0.11					**0.65**	0.16

Table S5. Data summary of the bone marrow of orthotopic and subcutaneous HNSCC bearing mice. Data is visualized in Figure 8. SD = standard deviation.

	Control						Subcutan						Orthotop					
	Day 7		Day 14		Day 21		Day 7		Day 14		Day 21		Day 7		Day 14		Day 21	
	**Mean**	SD	**Mean**	SD	**Mean**	SD	**Mean**	SD	**Mean**	SD	**Mean**	SD	**Mean**	SD	**Mean**	SD	**Mean**	SD
Leucocyte cells (% total)					**34.93**	0.09					**42.35**	4.58					**40.75**	4.95
Erythroid cells (% total)					**61.07**	1.07					**54.06**	4.09					**56.39**	4.79
B cells (×10^3)	**2982.90**	667.16	**3837.77**	1088.69	**4175.40**	1215.01	**3323.91**	1873.42	**4063.61**	2917.45	**3524.87**	2790.37	**3201.68**	1165.76	**3285.90**	402.20	**2996.99**	1514.13
Pro-Pre-B cells (IgM(lo)) (% B cells IgD(lo))					**61.58**	2.04					**69.58**	3.17					**75.28**	3.89
Immature B cells (IgM(med)) (% B cells IgD(lo))					**33.30**	0.93					**25.36**	3.11					**20.04**	2.84
Transitional B cells (IgM(hi)) (% B cells IgD(lo))					**4.24**	0.74					**4.90**	0.31					**4.39**	1.22

Table S6. Data summary of the thymus of orthotopic and subcutaneous HNSCC bearing mice. Data is visualized in Figure 9. SD = standard deviation.

	Control						Subcutan						Orthotop					
	Day 7		Day 14		Day 21		Day 7		Day 14		Day 21		Day 7		Day 14		Day 21	
	**Mean**	SD	**Mean**	SD	**Mean**	SD	**Mean**	SD	**Mean**	SD	**Mean**	SD	**Mean**	SD	**Mean**	SD	**Mean**	SD
T cells (×10^3)	**3156.56**	942.76	**2863.99**	70.63	**2218.02**	641.61	**2507.30**	1262.51	**2950.64**	1300.66	**2104.11**	1089.10	**2063.58**	890.89	**2764.92**	461.89	**2078.59**	1262.38
CD4+ T cells (×10^3)	**1294.27**	398.34	**1131.29**	93.32	**1317.55**	409.38	**1072.96**	559.91	**1363.76**	580.27	**1194.01**	633.98	**826.60**	359.12	**1186.10**	183.19	**1072.92**	638.63
CD8+ T cells (×10^3)	**1811.68**	512.63	**1660.82**	145.14	**868.90**	229.19	**1373.96**	681.09	**1534.16**	691.97	**872.78**	447.01	**1186.74**	533.01	**1504.12**	273.96	**963.29**	598.14
